# Integrating neuropsychoanalytic and neuropsychiatric perspectives into psychiatric clinical neuroscience curricula: a conceptual overview

**DOI:** 10.3389/fmed.2025.1712622

**Published:** 2026-01-05

**Authors:** Edward Miller, Michael Weightman, Andrew Amos, Steven Yeates, Fiona Wilkes

**Affiliations:** 1 Division of Psychological Medicine, The University of Auckland, Auckland, New Zealand; 2 Discipline of Psychiatry, University of Adelaide, Adelaide, SA, Australia; 3 Central Adelaide Local Health Network, Adelaide, SA, Australia; 4 School of Medicine and Dentistry, James Cook University, Townsville, QLD, Australia; 5 Australian Psychoanalytical Society, Sydney, NSW, Australia; 6 Academic Unit of Psychiatry and Addiction Medicine, The Australian National University School of Medicine and Psychology, Canberra, ACT, Australia

**Keywords:** clinical neuroscience, psychiatry training, medical education, neuropsychoanalysis, neuropsychiatry

## Abstract

**Objectives:**

Neuropsychoanalysis and neuropsychiatry are two rapidly advancing fields that can provide valuable additions to a clinical neuroscience curriculum in psychiatry and strengthen psychiatrists’ psychotherapeutic training and practice.

**Methods:**

This article provides an overview of key concepts of neuropsychoanalysis and neuropsychiatry, noting their common history in neurology, psychiatry and psychoanalysis. Several case vignettes are also provided to demonstrate how these concepts could be used to better understand specific psychiatric presentations.

**Results:**

Key concepts discussed include the shared history of neuropsychoanalysis and neuropsychiatry; philosophy of mind; the neuroscientific basis and function of different levels of consciousness; affective neuroscience; and the hierarchical network function of the brain.

**Conclusion:**

Neuropsychoanalysis and neuropsychiatry are subspecialties that have made important clinical and theoretical contributions to psychiatry. These insights could be used to inform the ongoing development of contemporary psychiatric clinical neuroscience curricula.

**Relevance to clinical practice:**

Psychiatrists can use this article to help with “bedside teaching” of junior staff, assist with patient formulation and psychoeducation, and ultimately inform an integrated pedagogical framework for the clinical training of psychiatrists.

## Highlights

1. Contemporary neuroscience research stemming from neuropsychoanalysis and neuropsychiatry can inform psychiatric clinical practice, teaching and training.
2. Neural network function, affective neuroscience and disorders of consciousness are implicated in psychiatric disorders.
3. Philosophy of mind and psychotherapy, including psychoanalysis, have more clinical psychiatric and neuroscientific relevance than ever.

## Introduction

Since the 1990’s, there has been an explosion of neuroscientific and philosophical advances in our understanding of brain and mind, with major implications for psychiatry. Psychiatry is increasingly considered a clinical neuroscience discipline, meaning that it deals with disorders that stem from disrupted brain networks and systems caused by underlying genetic, developmental, and environmental influences (1). However, the developments in neuroscience are technically complex and are occurring at a rapid pace. This makes them difficult to contextualize with other psychiatry curriculum elements that often do not progress as quickly, such as diagnostic criteria and nosology, some ethical principles (e.g., determinism and neuro-realism), mental health legislation, and psychotherapy training. This makes developing and refining a robust clinical neuroscience curriculum for psychiatry a difficult task (2, 3).

Consequently, the development of a clinical neuroscience curriculum for psychiatry training lags behind the neuroscientific progress, with many training programs having no such component, or there being wide variations in content when it is taught (4). For example, a review by Coverdale et al. identified only six examples of neuroscience curricula for psychiatry in the USA, which varied greatly in their number of lessons, class size, length, and curriculum content, with variations between neuroanatomy, neurology, pharmacology, interventional approaches and case formulations (2). Gopalan et al. (5) have attempted to standardize such a curriculum by proposing that the foundations of any clinical neuroscience curriculum should be based on the principle of learning about brain networks and basic connectivity features and functions, which would include (i) the fundamentals of neuroscience, such as neuronal structure, signaling and networking, and their development across the lifespan; (ii) a three-dimensional appreciation of neuroanatomy via brain models, neuroradiology, and brain dissection; and (iii) a focus on how neural dysfunctions may manifest with affective, behavioral, and cognitive phenotypes. There are also many and varied curriculum resources available online, including through the National Neuroscience Curriculum Initiative^1^, the Society for Neuroscience^2^, Genes 2 Cognition Online^3^, and the Federation of European Neuroscience Societies^4^.

Neuropsychoanalysis and neuropsychiatry represent the integration of neuroscience with the theory and practice of psychoanalysis and psychiatry, respectively. Neuropsychoanalysis, in particular, arises from the direct incorporation of neuroscientific methods and concepts into psychoanalysis, forming a distinct but complementary interdisciplinary field. It follows that highlighting key concepts from these fields would greatly enhance the development of a clinical neuroscience curriculum for basic psychiatry training. Moreover, discoveries from neuropsychoanalysis and neuropsychiatry can deepen psychiatrists’ understanding of the physical, cognitive, and emotional mechanisms that operate during successful and unsuccesful psychotherapy, helping them to become more informed psychotherapists by leveraging their unique training in both contemporary mind and brain science.

This article aims to provide a short conceptual overview of the key ideas from neuropsychoanalysis and neuropsychiatry which could be used to inform and standardize a clinical neuroscience curriculum for psychiatrists. This includes highlighting the origins of both neuropsychoanalysis and neuropsychiatry in psychoanalysis, neurology and early psychiatry; noting the importance of philosophy of mind, psychoanalysis, consciousness, and affective neuroscience for neuropsychoanalysis; and discussing the importance of hierarchical brain network function and scope of disorders and treatments for neuropsychiatry. The paper will not define or outline a curriculum, although it will describe a number of case vignettes that could be used for teaching purposes. The overview of concepts described here could be used for future curriculum development, such as in lecture material, online teaching modules, bedside teaching, psychotherapy training for psychiatrists, and case formulation and discussion.

## Neuropsychoanalysis

The term “*neuropsychoanalysis*” was coined in 1999 by South African neurologist Mark Solms to describe the integration of neuroscience with psychoanalytic theory and practice. Neuropsychoanalysis extends psychoanalysis through neuroscientific investigation while maintaining continuity with its broader depth psychological heritage.

Solms’ research on stroke patients with cortical deficits had shown that brain damage altered the phenomenology of their dreams (6). He subsequently described the origin of neuropsychoanalysis as psychoanalytic work on neurological patients, and explored the implications for psychoanalytic psychotherapy (7). Neuropsychoanalysis has been described as “an attempt to integrate psychoanalysis into the neurosciences, as a member of the family of neurosciences—but the one that studies the mental apparatus from the subjective point of view” (6) (p141). “Therefore, neuroscience and psychoanalysis have been viewed as two perspectives of the same ‘unknowable thing in nature” (the reality behind the subjective mind and objective brain) which should not seek to necessarily solve the riddle, but rather to “straddle the gap” (8). The field of neuropsychoanalysis also has the potential to bridge theoretical gaps between different psychoanalytic schools, which have often remained insular and separate (9). It is conceptualized as an attempt to link the corpus of psychoanalytic theory with the neurosciences, and vice versa (6).

Neuropsychoanalysis draws contributors from a wide range of disciplines, including neurology, clinical and research psychology, psychiatry, psychoanalysis, and research neuroscience. Currently, the field remains mostly theoretical, technical, and research-orientated, with no established clinical training pathway as of the time of writing. This absence largely reflects the fact that neuropsychoanalysis functions primarily as an integrative framework rather than a standalone clinical discipline.

## Dual aspect monism and neuropsychoanalysis

While Baruch Spinoza’s dual aspect monism (DAM) has been a dominant philosophical paradigm in the development of neuropsychoanalysis, it has also functioned as a solid foundation for proposed extensions like transcendental materialist, and as a launching pad for departures like post-Kantian thought. For example, Talvitie and Ihanus highlight that DAM may sometimes overstate ontological unity at the expense of epistemic complexity (10). Others propose alternate frameworks for conceptualizing neuropsychoanalysis; transcendental idealism may better accommodate the constitutive role of subjectivity in mental phenomena (11), while Immanuel Kant’s transcendental philosophy is also relevant, particularly his claim that the mind actively organizes experiences rather than merely receiving external information (6). Contemporary predictive processing and Free-Energy models (e.g., Friston; Solms) align in form, (though arising from different intellectual traditions), emphasizing that perception involves active inference and hypothesis-testing rather than passive reception of reality. A fuller discussion of predictive processing and its clinical relevance appears in the neuroscience section below.

Spinoza’s DAM, articulated in his 1600s opus *Ethics*, presaged much of the modern neuroscientific investigation of affects and emotions (a field now called affective neuroscience), which also influenced Freud (6). Spinoza formulated DAM in response to his contemporary René Descartes’s theory of dualism (12). Descartes postulated that mind and brain are separate entities, which accorded with prevailing religious dogmata including the existence of immortal souls. For Descartes, the soul resides in the pineal gland, which was its interface to the body, but both were fundamentally different entities, and the soul was therefore theoretically able to persist beyond death. Spinoza disagreed. In *Ethics*, he described mind and brain as two inseparable attributes of a single underlying Substance, each expressing the same reality under different modes; a position referred to as DAM because the Substance (monism) has two (dual) aspects. To Spinoza, mind and brain are neither completely representative or epiphenomenal to the other, but merely occupy opposite and interrelating aspects. Crucially, neuropsychoanalysis adopts this framework in a non-reductive sense: mind and matter are regarded as complementary expressions of a single underlying reality, rather than one being derived from or reducible to the other. This distinguishes neuropsychoanalytic theory from either physicalist reductionism or Cartesian dualism and situates it within a co-instantiated monist ontology. However, this position was historically overshadowed by Cartesian dualism and has only recently re-entered modern vocabulary. Although some authors have drawn parallels between DAM and positions such as transcendental materialism and emergentism (e.g., Dall’Aglio), these frameworks represent distinct ontological commitments. DAM posits an ontologically neutral foundational substance, whereas transcendental materialism and emergentism retain forms of physicalist monism in which mind is dependent on or emerges from matter. These debates reflect ongoing efforts to clarify the philosophical foundations of neuropsychoanalysis (8).

A central clinical implication of ontological pluralism is the cultivation of epistemic humility. If multiple metaphysical frameworks can meaningfully account for mind–brain relations, then no single explanatory model should be held as complete or final. This philosophical stance translates clinically into the capacity to tolerate uncertainty and to maintain an attitude of “not-knowing” regarding patients’ inner worlds—a skill psychoanalysis has long emphasized. This humility supports curiosity, receptivity, and the patient-led emergence of meaning rather than premature theoretical closure. Thus, rather than being abstract, engagement with philosophy of mind directly shapes psychotherapeutic stance and reflective practice in psychiatric training. The following Table 1 provides a representative overview of major mind–brain ontologies historically influential in psychiatry and neuropsychoanalysis. It is not exhaustive. Recent literature has highlighted additional metaphysical options—including idealism, transcendental idealism, and panpsychism—which we briefly introduce here and discuss in the main text; however, for clarity and pedagogical focus this Table 1 highlights the dominant positions most commonly referenced in clinical neuroscience and psychodynamic theory.

**TABLE 1:** Overview of the main mind–brain philosophies [adapted from Cheniaux and Lyra (12)].

Philosophy	Sub field	Key exponents	Explanation
Dualism	Interactionism	Descartes	Mind and matter are distinct and independent, though they exert causal effects on each other
	Psychophysical parallelism	Leibniz	Mental and physical phenomena are parallel, simultaneous, and correlated, but not causal
	Epiphenomenalism	Huxley	Mental events are by-products of physical processes (e.g., brain activity, sensory inputs) and lack causal efficacy
Non dualism (monism)	Analytical behaviorism	Ryle and Hempel	Mental concepts are analyzed as behavioral dispositions; explanation avoids inner mental entities
	Reductive materialism	Smart; Place; Armstrong	Mental states are type-identical with physical brain states
	Functionalism	Lewis; Putnam; Fodor; Dennett	Mental states are individuated by causal/functional role; mind installed over hardware (wetware)
	Eliminative materialism	Paul and Patricia Churchland; Stich	Common-sense “folk psychology” posits entities that may not exist; mature neuroscience will replace them
	Dual aspect monism	Spinoza	Mind and brain are two aspects of a single underlying Substance; neither reduces to the other
	Transcendental materialism	Dall’Aglio	A physicalist monism in which mind emerges from matter; sometimes compared with DAM but ontologically distinct
	Idealism	Berkeley; Hegel; Schopenhauer	Mind/consciousness is primary; physical reality depends on mind
	Transcendental idealism	Kant	Experience is structured by a priori forms; mind and world are co-constituted
	Panpsychism	James; Strawson; Goff	Mind-like properties are fundamental and ubiquitous in nature; consciousness exists on a continuum across levels of physical complexity

## The key conceptual areas of neuropsychoanalysis

### Neuroscience of unconscious mental processes

Eric Kandel suggested that the link between Freud’s topographic and structural models and memory systems would be a fruitful area for collaboration between psychoanalysts, biologists, and neuroscientists: Different layers of the unconscious (including the preconscious unconscious and unconscious proper) might be linked to different memory systems and corresponding brain regions and networks, such as the prefrontal cortex (9). The work of Solms, Panksepp, and others in this area can also be linked to Tulving’s layers of anoetic, noetic, and autonoetic consciousness, which correspond to procedural, semantic, and episodic memory systems (13). Dream psychology and dream physiology also have thorough neuroscientific descriptions which can be linked to Freudian dream theory. An example is Solms’ demonstration that dream imagery is instantiated in the medial prefrontal and parietal areas rather than brainstem nuclei (14). Solms also suggested that neuroscientific processes can help to test, update, and refine Freudian theory.

For example, Solms’ reinterpretation of Freud’s topographical model, grounded in affective neuroscience, reverses the classical psychoanalytic assumption that the ego is conscious and the id unconscious (15). Instead, Solms argues that affect—the core of the id—is inherently conscious, because feelings are the primary means by which the organism registers biological value and motivational salience. By contrast, the ego, as a cognitive and predictive system, operates largely unconsciously or pre-consciously, consistent with contemporary hierarchical models of cognition and predictive processing. This model places emotional feeling, rather than perceptual representation or symbolic thought, at the core of conscious awareness. Neurobiologically, this view aligns with Jaak Panksepp’s affective neuroscience framework, which locates the foundations of core emotional consciousness in brainstem networks, particularly the periaqueductal gray (PAG) and related subcortical structures (16). Cognition-based self-models, associated with ego functions, arise secondarily in higher cortical networks and are therefore not the primary source of consciousness but rather systems that interpret and regulate affective states. The use of neuroimaging methods may also help to unmask unconscious processes in other psychoanalytic concepts such as mourning (17), or psychoanalytic perspectives on confabulation (18).

Predictive processing provides a mechanistic account of unconscious inference and affect-driven perception, grounded in the Free-Energy principle (FEP) that was introduced by Karl Fritson in his 1990’s brain imaging work (19). The FEP has been described as the new “royal road in the dialogue between neuroscience and psychoanalysis, the bridge between mind and brain” (20) (p3). The FEP analogises the mind–brain system to any other biological system that requires adaptation to its environment to survive. It considers the brain as a hierarchically constructed inferential machine containing many interconnected large-scale networks. These hierarchical networks optimize the prediction, representation, and construction of increasingly complex lower order mental and sensory information to more accurately interpret and predict the meaning of the internal and external environment (19). This “minimizes” the amount of free energy (statistical uncertainty or surprise) available in the mind and brain, and reduced prediction error, which can also be understood as reducing the statistical complexity of the information contained in the brain. This means the mind–brain can never “know” the world directly, but only perceive it by predicting what it could be like, then testing that against new information.

While the FEP has been broadly applied across cognitive neuroscience, it has particular relevance to neuropsychoanalysis. Solms (21) has argued that the FEP provides a formal solution to the “hard problem” of consciousness by grounding subjective feeling in homeostatic affective processes. In this account, consciousness originates from affective valuation systems concerned with survival and biological regulation, rather than from cortical cognitive representations. This interpretation aligns the FEP with Pankseppian affective neuroscience and the neuropsychoanalytic model in which affect constitutes the core of conscious experience and cognition functions primarily to predict, symbolize, and regulate affective demands. Thus, the FEP offers a computational and biological bridge between Freudian drive theory, affective neuroscience, and contemporary models of predictive processing in the brain (21, 22).

It is important for survival that the mind–brain tries to predict reality as accurately as possible. Within predictive-processing frameworks, perception is understood as the brain’s best inference about the causes of sensory inputs, based on prior expectations and incoming signals. As Seth notes, ordinary perception can be described as a “controlled hallucination,” meaning that hallucination-like processes are inherent to conscious experience rather than intrinsically pathological (23). Psychopathology is theorized to arise when prediction errors and prior beliefs become maladaptively weighted, such that excessively strong priors dominate sensory evidence or vice-versa (24, 25). Under this account, clinical hallucinations may reflect aberrant precision-weighting of priors, not a categorical departure from normal perception, but a quantitative disruption of the same generative process. However, these computational accounts describe *how* symptoms may arise at a mechanistic level, rather than identifying their origins. The development of maladaptive priors is deeply shaped by early relational experience, attachment, trauma, and psychosocial context (26). This formulation aligns with neuropsychoanalytic views in which unconscious affective drives shape predictive models, meaning that disruptions in affective regulation may influence perceptual inference.

**FIGURE 1:**
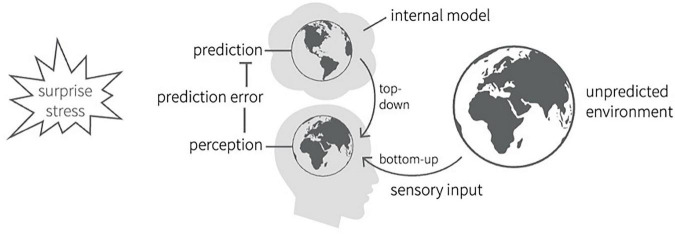
Predictive coding in the Bayesian brain [adapted from Haker et al. (60)] used with license CC BY 4.0. In this figure, the internal prediction may be that someone is expecting the weather to be sunny and not raining. Upon receiving sensory information that it might be raining outside, there is now a mismatch or error between the predicted world and the sensory world, which creates statistical surprise and stress. The internal working model must adapt based on the new data.

The basic model summarized in Figure 1 is similar to Freud’s original topographic model of primary and secondary processes (19). The primary process generates unconscious fantasies, wishes, and desires generated by the id (bottom-up processing), which are countered by the (top-down) ego’s affinity for contrasting these impulses against the incoming sensory information and the “reality” of the external world. According to Jim Hopkins, the FEP overlaps with psychoanalysis in understanding emotional conflict and trauma; understanding the function of memory consolidation and reconsolidation in dreaming; and identifying the role played by the mind–brain’s tendency to predict reality in order to reduce statistical complexity and “buffer” against excessive statistical surprise, in the origins of a range of mental disorders such as schizophrenia (27). The claim that the mind–brain does not directly apprehend reality, but instead infers it through predictive modeling, reflects an epistemological stance aligned with Kantian and contemporary predictive-processing accounts. This does not contradict the ontological position of dual-aspect monism adopted in neuropsychoanalysis. Rather, DAM provides a framework for understanding mind and brain as complementary aspects of a single underlying reality, while predictive epistemology clarifies that this reality is encountered indirectly through affectively-weighted inferential processes. Thus, neuropsychoanalysis can accommodate both ontological non-reductionism and epistemic humility about the limits and structure of perception.

### Neurobiology of infancy, development and affective neuroscience

Psychoanalytic accounts have long emphasized that the infant mind develops in the context of early relational experience. Freud highlighted innate motivational drives and maturational processes, proposing developmental stages built on endogenous instinctual pressures and their regulation (28). Subsequent developments, particularly Bion’s work, expanded this view by emphasizing the internalization of early object relations and the centrality of the caregiver–infant dyad in shaping emotional and representational capacities. Bion conceptualized this process through the functions of containment, reverie, and alpha-function, whereby the caregiver metabolizes and transforms the infant’s raw emotional states into tolerable and thinkable experiences (29). This relational process supports the emergence of mentalisation, affect regulation, and symbolic thought. Modern affective neuroscience and developmental neurobiology—including research on attachment, social buffering, and right-hemisphere maturation—provide convergent empirical grounding for these psychoanalytic formulations (19). This aligns with Schore’s demonstration that early secure attachment drives right-hemisphere development and lifelong affect regulation capacities (30). Trevarthen’s work similarly shows that infants possess innate capacities for intersubjectivity and affective communication, providing a neurodevelopmental foundation for Bion’s model of containment and mentalisation (31). More recently, the longitudinal build-up of unconscious representations of visceral states and their representations in thought has been linked to activity in the posterior and middle insula and the default mode network (32).

Kandel discussed this build-up in terms of psychological determinacy, causality, and development, including the role of early life experience and predisposition in the development of psychopathology, and linked this to Freudian constructs like signal anxiety (9). Lehtonen suggested that neurobiological and proto-psychological processes during the perinatal period can be linked to psychoanalysis (33). For example, the impact of early attachment and caregiving relationships can be mapped to specific biochemical processes and the molecular-genetic mechanisms of neuronal synapse formation (34). These processes are “formative for the early organization of the mind and also create preconditions for non-verbal, unconscious communication and therapeutic interplay” (33) (p14). Recent developmental neuroscience suggests that non-declarative memory and rudimentary self-organization capacities are present from birth, consistent with Kleinian proposals of early ego functions. For example, newborns show differential processing of familiar versus unfamiliar faces (35), indicating proto-representational and regulatory capacities. Mellor (13) argues that if such early mnemonic functions are considered part of ego activity, this provides neuroscientific support for psychoanalytic models proposing a nascent ego from birth. These findings remain interpretative, linking empirical data with psychoanalytic theory rather than offering direct proof (13).

**FIGURE 2:**
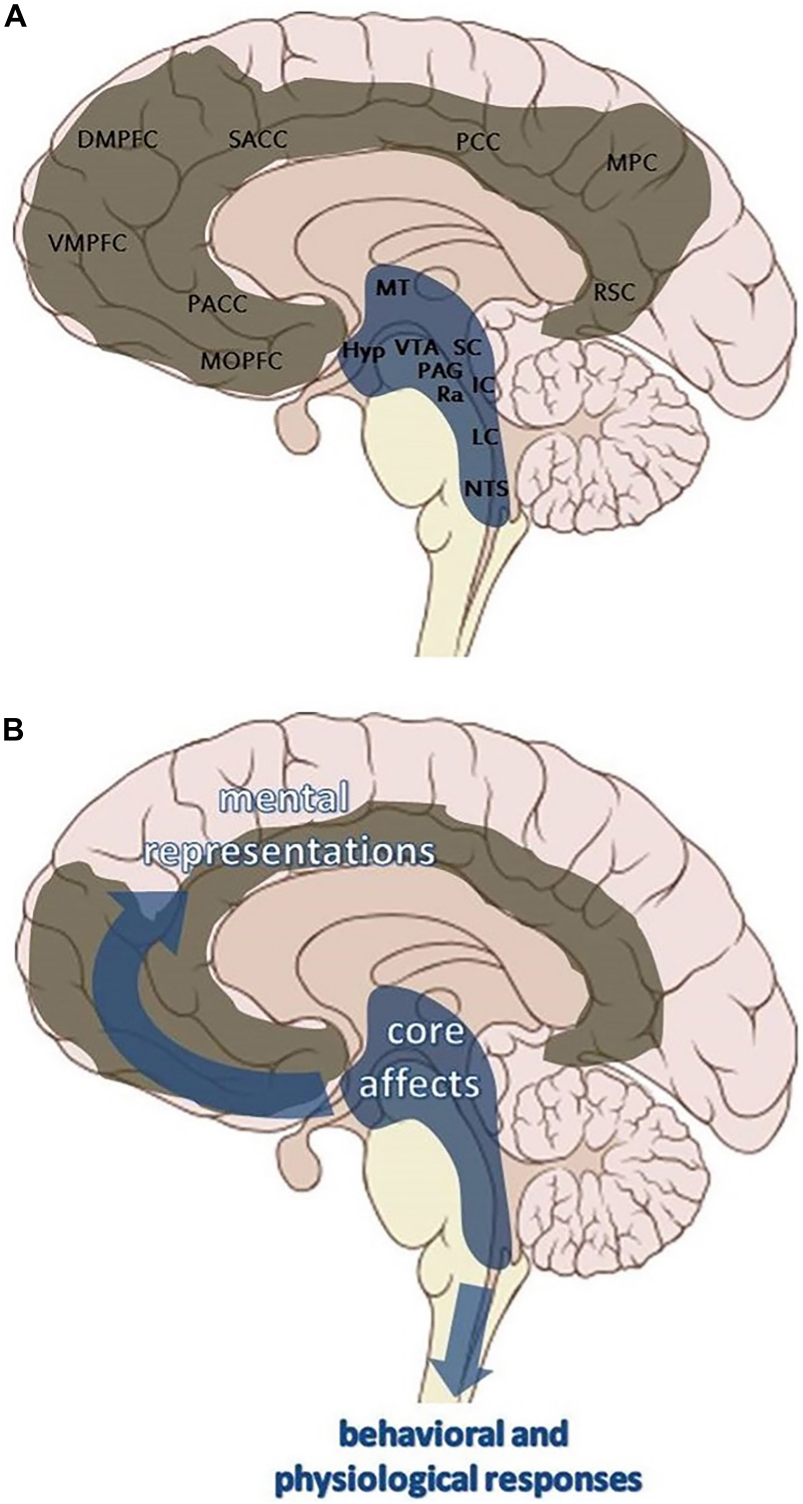
**(A)** Schematic illustration of the ascending and descending pathways where core affective neurodynamic states originating within subcortical midline structures (SCMS) are transmitted (61). **(B)** Schematic illustration of the midline structures of the brain. Subcortical midline structures (in blue): Hyp, hypothalamus; IC, inferior colliculus; LC, locus coeruleus; MT, mediodorsal thalamus; NTS, nucleus tractus solitaries; PAG, periaqueductal gray; Ra, raphe nuclei; SC, superior colliculus; VTA, ventral tegmental area. Cortical midline structures (in gray): DMPFC, dorsal medial prefrontal cortex; MOPFC, medial orbital prefrontal cortex; MPC, medial parietal cortex; PACC, pre- and subgenual anterior cingulate cortex; PCC, posterior cingulate cortex; RSC, retrosplenial cortex; SACC, supragenual anterior cingulate cortex; VMPFC, ventral medial prefrontal cortex (61) used with license CC BY 4.0.

These psychoanalytic models correspond to contemporary affective neuroscience findings that primary emotional consciousness arises from subcortical affective systems, with higher-order cognitive functions emerging relationally and developmentally (16). Panksepp’s description of mammalian affective systems has been described by Solms as a translation of Freudian drive theory based on more precise research (19). Panksepp described nested BrainMind hierarchies, where primary process emotion systems—genetically endowed affects in the deep subcortical regions of the brain—become shaped and molded by learning and conditioning in the midbrain, before being represented in symbolic and tertiary-level thought in the neocortex (Figure 2A) (16). This “bottom-up” process is then regulated by “top-down” neocortex inputs in what is referred to as a two-way or circular causation process of representation and learning (16). Panksepp’s affective systems are summarized in Table 2 and Figure 2B. Panksepp argues that the seat of consciousness (the Self), as it relates to and intertwines with affect and drives, is now increasingly established to be subcortical rather than cortical (16).

**TABLE 2:** Affective systems as described by Panksepp and Biven (16) [adapted from Penner and Stoddard (36)].

Affective system	Key brain regions	Associated emotional phenomenon
LUST	Corticomedial amygdala, BNST, preoptic hypothalamus, VMH, PAG	Erotic feelings, jealousy
CARE	Preoptic area, anterior cingulate, VTA, PAG, BNST	Love, attraction
PANIC	Anterior cingulate, BNST, dorsomedial thalamus, PAG	Separation distress, guilt, embarrassment
PLAY	Dorsomedial diencephalon, parafascicular area, PAG	Joy, playfulness
FEAR	Central and lateral amygdala to medial hypothalamus and dorsal PAG	Anxiety, worry
RAGE	Medial amygdala to BNST, perifornical hypothalamus to PAG	Anger, irritability
SEEKING	Nucleus accumbens, VTA, mesolimbic and mesocortical outputs, lateral hypothalamus, PAG	Interest, frustration, craving

BNST, bed nucleus of stria terminalis; PAG, periaqueductal gray; VMH, ventromedial hypothalamus; VTA, ventral tegmental nucleus.

Although, on current available evidence, affective neuroscience locates core consciousness in subcortical structures such as the PAG (16), this neurobiological localization should not be conflated with a reductive materialist ontology. From a dual-aspect monist perspective, neural substrates constitute one aspect of a single underlying reality in which subjective affect and brain processes co-instantiate one another. As Goldreich (11), Talvitie and Ihanus (10) note, neuropsychoanalysis must guard against implicit physicalism, while acknowledging that the neural implementation of affective consciousness need not commit the theory to materialist monism. Rather, neurobiological findings are interpreted here as the physical correlate of affective experience, consistent with a non-reductive dual-aspect formulation. This position parallels Kantian and predictive-processing views wherein the brain generates models of experience, without implying that experience reduces to neural computation.

### Neuroscience in relation to psychoanalytic practice

Neuropsychoanalysis uniquely integrates psychoanalytic and neuroscientific perspectives to enrich the conceptual foundations of psychotherapy, rather than to dictate technique or mechanistic explanations of change. It is important to clarify that linking psychoanalytic constructs to neural processes is not intended to suggest that neuroscience directly informs moment-to-moment psychoanalytic technique. Rather, such links offer a conceptual bridge that can deepen clinicians’ reflective understanding of affect, motivation, and unconscious mental processes, without replacing phenomenological and relational approaches. We argue that considering neuroscientific correlates may add value to psychoanalytic practice through enriching training, formulation, and theoretical coherence, provided such insights are understood as complementary rather than prescriptive. We acknowledge, as others argue, that neuroscience does not and cannot substitute for the lived emotional and symbolic dimensions of psychoanalytic work (37, 38). Our stance aligns with Yovell et al. neurobiological insights may enrich theoretical coherence, training, and reflective understanding, while technique remains grounded in relational experience and symbolic process (7). Neuroscientific perspectives can potentially deepen clinicians’ conceptual insight into affect regulation, motivational systems, and unconscious processing; strengthen integrative formulation by linking developmental, affective, and neural processes; and support reflective practice by grounding therapeutic principles in models of emotional and predictive functioning. In this sense, neuroscience informs how we think about the mind and suffering, rather than how we conduct moment-to-moment interventions, complementing rather than replacing interpersonal and symbolic modes of working.

Multiple studies have researched the neuroscience of psychoanalytic concepts, including transference and countertransference, defense systems, free association, and dreaming and trauma (39). Indeed, transference and countertransference are regarded as cornerstones of psychoanalytic and psychodynamic psychotherapies, as well as having broad applicability across a range of psychotherapeutic modalities. Transference in particular has been defined as “a tendency in which representational aspects of important and formative relationships (such as with parents and siblings) can be both consciously experienced and/or unconsciously ascribed to other relationships” (40) (p392). In this sense, transference relates to the implicit relational schema and dynamic pattern matching of a patient invoked in therapy, based on a priori learning and memory formation. Activation of these a priori emotional processes in therapy is key to psychic change, part of which involves activation and remodeling of declarative and procedural memory networks, with associated neural correlates in the superior and inferior frontal gyri and in the putamen (39). In other words, transference is a premature automatisation of which interpretation helps to render into working memory. Neuroscience does not seek to instruct clinicians *how* to interpret transference or conduct moment-to-moment therapeutic work. Rather, it clarifies *why* transference phenomena arise, *for whom* they are especially salient, and *how* early internalized affective templates shape perception and expectation. Understanding transference as the brain’s predictive modeling of interpersonal experience—shaped by prior relational learning and affective memory—can strengthen clinicians’ appreciation of its developmental origins, its universality, and its function as an attempt to regulate uncertainty and affect. These neuroscientific accounts enrich theoretical formulation and training without replacing lived clinical intuition or symbolic engagement.

Understanding the neuroscience of similar therapeutic processes, such as automatic mirroring and reflective functioning, helps to explain clinical phenomena such as therapeutic alliance and change, since the neural motor networks of psychotherapy patients may automatically mirror the motor movements of the therapist, which then may impart effects on the neuronal systems in the brain of the patient, including through the function of motor neurons (33). Evidence relating to mirror neuron systems and interpersonal resonance does not imply that therapists require neuroscientific knowledge to form therapeutic alliances or attune to patients. Rather, such findings demonstrate that the capacity for empathy, emotional attunement, and affective mirroring is biologically instantiated and developmentally shaped. In psychiatric and psychotherapeutic education, these neuroscientific perspectives can help integrate relational and biological discourses, affirming that the therapeutic relationship is grounded in evolved neural mechanisms of social connection rather than being “merely subjective” or intangible. In this sense, neuroscience supports the legitimacy and conceptual integration of relational work within medical training, without providing technical guidance or replacing the lived interpersonal process.

A previous review suggested that psychotherapy has measurable effects on brain structures (41). One mechanism of action for this may be that psychotherapy causes changes to the serotonin-driven neuronal networks in the brain of subjects with depression (42). Jeremy Holmes hypothesizes that some psychotherapeutic interventions, such as psychoanalytic psychotherapy and mentalization-based treatment, “work” because of their propensity to bind free energy, (i.e., reduce unpredictability or minimize prediction error), which enhance Bayesian inference and allow “experience and feeling states to be “metabolized” and assimilated” (26) (p1).

Psychoanalytic work addresses early relational experience as it is revived and transformed within the transference relationship, and such experiential change is necessarily instantiated in corresponding neural processes. In a dual-aspect monist framework, these are not separate pathways but two inseparable aspects of the same therapeutic transformation. Holmes has analogised this process to be “qualitatively akin to neurosurgery,” with the mind–brain as the operative target (43) (p24).

Consider a patient with early attachment trauma who presents with intense fears of abandonment and a tendency to expect rejection in relationships. Psychoanalytically, such a presentation may be understood as the activation of internalized object relations and transference expectations shaped by early caregiving. Neuroscientific findings complement this by showing that early relational adversity can sensitize limbic and brainstem affective circuits, alter stress-regulation systems, and bias threat-prediction networks (e.g., amygdala–PAG–hypothalamus pathways), leading the individual to over-predict interpersonal danger (16). Integrating these perspectives can enrich formulation by linking early relational patterns, affective dysregulation, and defensive strategies to underlying motivational and neurobiological processes. It is also acknowledged that the nature of the clinical relationship, rather than any specific theoretical framework, is the best predictor of treatment outcome (44).

## Critiques for and against neuropsychoanalysis

Neuropsychoanalysis has drawn both strong support and significant critique. A central concern is that, despite its dual-aspect monist foundations, it may implicitly slip into biological reductionism if neural explanations are prioritized over subjective meaning (37, 38). From this perspective, a valid neuropsychoanalysis must maintain genuine bi-directionality between mind and brain: neural mechanisms may correlate with and illuminate psychoanalytic processes, but they do not replace hermeneutic inquiry or symbolic understanding. As Dall’Aglio (8) argues, an integrative approach requires a balance between subjective experience and objective neuroscience, rather than a token appropriation of scientific concept.

A further critique concerns the possibility that clinicians may unconsciously adopt biological explanations defensively when encountering clinical uncertainty or resistance. For instance, a therapist may be tempted to challenge a patient’s recollection of birth trauma based on neuroscientific memory constraints rather than staying with the symbolic meaning of the experience (37). Similarly, prescribing medication may at times function as a countertransference defense against anxiety rather than solely addressing biological dysregulation. These concerns highlight the importance of maintaining psychoanalytic sensitivity to transference, countertransference, and meaning—while recognizing that neuroscientific knowledge does not inherently improve technique and must be used judiciously.

This defensive function is not limited to clinicians. Reflexively, as scholars we may also be tempted to invoke neuroscience to manage epistemic uncertainty, solidify theoretical positions, or alleviate discomfort in the face of unconscious processes or conceptual ambiguity. Acknowledging this possible tendency reinforces the necessity of epistemic humility and negative capability: the capacity to tolerate not-knowing and complexity rather than seeking premature explanatory closure. This stance is consistent with the psychoanalytic tradition of thinking about thinking, and aligns with the pluralistic ontological position adopted earlier in this paper.

Another critique relates to the diversity of psychoanalytic traditions. Przybyła notes that neuropsychoanalysis is sometimes perceived as overly aligned with Freudian and object-relations frameworks, to the exclusion of other influential traditions such as Lacanian and phenomenological approaches (45). Additionally, others argue that the very term *neuropsychoanalysis* symbolically privileges neuroscience (46). Blass and Carmeli (37) further contend that psychoanalysis fundamentally concerns the hermeneutic study of unconscious meaning, whereas neuroscience concerns physical processes, and the two should not be conflated. In contrast, Fonagy, alongside Yovell et al. argue that interdisciplinary dialogue is not only possible but necessary for a contemporary understanding of the mind, provided neither paradigm is subordinated to the other (7, 47).

Within psychiatry, clinicians are in a unique position to engage with both neuroscience and psychotherapy. A clinical neuroscience curriculum that includes affect, subjectivity, and relational experience may support an integrative understanding of mind–brain processes (3). Importantly, neuroscientific literacy does not directly produce core psychotherapeutic skills—such as attunement, presence, tolerating uncertainty, or maintaining a reflective stance—but can legitimize these humanistic principles within a biomedical training context. For example, attachment research and affective neuroscience can help clinicians appreciate how early relational experience shapes affect regulation and interpersonal prediction, complementing experiential learning rather than replacing it.

Similarly, linking psychoanalytic concepts such as transference and countertransference to neuroscientific models of affective learning, predictive processing, and social cognition can enrich theoretical coherence. For instance, transference may be conceptualized as the activation of relational prediction models shaped by early attachment experiences, instantiated in limbic and prefrontal circuits, while countertransference can be understood through the lens of embodied affective resonance and the therapist’s own predictive systems. These correspondences do not dictate technique, but they can deepen formulation, support interdisciplinary dialogue, and strengthen appreciation of the biological grounding of relational processes.

While neuropsychoanalysis offers a promising interdisciplinary framework, its development remains constrained by several limitations. Empirical research linking psychoanalytic constructs with neurobiological processes is still emerging, methodological challenges persist in operationalising dynamic concepts without reductionism, and debate continues regarding appropriate levels of explanation and evidentiary standards. Future work would benefit from deeper engagement with diverse psychoanalytic traditions, rigorous conceptual clarification of key constructs, and longitudinal or process-oriented research designs that trace experiential, relational, and neural change in parallel. Crucially, continued dialogue between clinicians, philosophers, and neuroscientists is required to ensure that the field evolves without collapsing into either biological reductionism or purely hermeneutic isolation. Such interdisciplinary collaboration may strengthen our capacity to understand and alleviate human suffering while maintaining epistemic humility and pluralism. In light of these debates, a key question becomes how neuropsychoanalysis can be integrated into psychiatric and psychotherapeutic education in a way that honors both its scientific grounding and its commitment to subjectivity, complexity, and relational depth.

## Neuropsychiatry

While neuropsychoanalysis primarily contributes at a conceptual level, neuropsychiatry often yields clear operational implications for diagnosis, treatment planning, and clinical decision-making. Taken together, these perspectives may offer complementary routes to understanding and responding to mental suffering in psychiatric practice.

### Historical origins

Neuropsychiatry has its origins in Russian psychologist Alexander Luria’s key advances in establishing the brain science underpinning certain complex psychological functions (48). Luria began his career in the 1920’s with a deep interest in psychoanalysis, entering into correspondence with Freud in 1922 and founding a psychoanalytic society (49). By the end of his career in the late 1970’s, he had formulated mind and brain as hierarchical functions with a complex structure and genesis, and subserving overlying psychological functions that, unlike neurology, emerge developmentally from these neural systems but cannot be precisely localized except for some key elementary components (50).

Luria’s interest in psychoanalysis influenced him to link personal experience and development with mind and brain function, leading to the emergence of the field of “neuropsychiatry” in the 1980’s. A 1985 symposium in Paris marking the 100^th^ anniversary of Gilles-de-la-Tourette syndrome is cited as a turning point in the acceptance that a disorder could have both biological and psychological components, rather than being exclusively either neuroanatomical or functional (51). With increased recognition of neurological aetiologies of psychiatric conditions, the British Neuropsychiatry Association (BNPA) was established in 1987, and the American Neuropsychiatric Association (the ANA, and now ANPA) was established in 1989 (51).

### Contemporary classification

Neuropsychiatry is now often classified as a hybrid discipline straddling the border between psychiatry and neurology (52). Neuropsychiatry is sometimes seen as the psychiatry of brain disorders, i.e., that “neuropsychiatrists work with patients with mental disorders which in most cases originate from a brain malfunction” (53). Broadly, neuropsychiatry is said to emphasize “cognitive” functions such as episodic memory, visual attention, executive control, and visually guided action (54). Neuropsychiatry remains at present a subspeciality of psychiatry, and unlike neuropsychoanalysis, has its own training and qualification pathway in many psychiatric postgraduate colleges (55).

### Systemic–dynamic localization theory

Given neuropsychiatry’s focus on broad cognitive domains, Northoff suggests that neuropsychiatry should focus on a “systemic–dynamic” localization. This means that phenotypic function is attributed to a network of hierarchical interconnections which contribute to pluripotential functional or phenotypic systems, rather than anatomy (50). Northoff suggests this network may incorporate “resonant oscillator circuits” associated with a degree of “neuronal integration.” An example is the association between subthalamic beta-band activity and Parkinson’s disease (56).

Northoff describes neuronal integration as “the coordination and adjustment of neuronal activity across multiple brain regions,” which is “considered necessary for a complex function to occur, such as emotion or cognition” (50) (p230). Similarly, Friston and Price have distinguished between functional connectivity, the outcome of remote neurophysiological events (including the way these are mediated by other factors), and effective connectivity, which describes “the [direct] influence that one neural system exerts over another, either at a synaptic… or population level” (57) (p277). On a micro-scale, connectivity may be impacted by the number of synapses, dendrites, or neurotransmitter factors; while on a meso-scale, there may be columns of local connections; and on a macro-scale, there may be links between neuronal populations by pathways from different regions of the brain (57). Combinations and degrees of interaction between all three levels can then give rise to their respective functional connectivity. Northoff has described how these processes may relate to traditional psychiatric and neuropsychiatric disorders, such as: top-down modulation and its role in post-traumatic stress disorder; reciprocal modulation in depression; modulation by reversal in phobias; and modulation by functional unity in catatonia (50).

### Scope of disorders and interventions

While neuropsychiatry focuses on disorders with identifiable brain-based contributions and corresponding biomedical or rehabilitative interventions, this does not imply a reduction of psychological or relational processes to neural substrates; rather, it highlights one complementary mode of understanding within psychiatric care. Neuropsychiatry addresses multiple disorders where cognitive, behavioral, or affective disturbance results directly from brain changes (52). It has a reasonably well-defined set of treatments, including: neurostimulation, neuromodulation, neuropsychiatric rehabilitation (including measures to enhance neuroplasticity), and pharmacotherapy. Electroconvulsive therapy remains the main neurostimulation modality, but has been joined by vagus nerve stimulation (VNS), transcranial direct current stimulation (tDCS), and transcranial magnetic stimulation (TMS) (52). Deep brain stimulation (DBS) is another important modality of treatment for neuropsychiatric conditions, particularly for Tourette’s syndrome, obsessive-compulsive disorder, and depression. In the future, other psychiatric treatments such as gene therapy, stem cell use, and brain implants may involve direct brain intervention and could be regarded as neuropsychiatric (58).

### Rehabilitation, neuroplasticity and biomarkers

Neuropsychiatric rehabilitation is a relatively new field that originated in the treatment of traumatic brain injury, but is being researched to treat a range of disorders with neurocognitive effects, such as the dementias and schizophrenia, by addressing dysfunctions of neuroplasticity and neurogenesis (52). Similarly, cognitive stimulation and training, such as “executive exercises” and memory training, are being researched as a means to improve cognitive function, particularly in the prevention of dementia (52). Moreover, a large focus of neuropsychiatric research is in the field of biomarkers and other “-omics” discoveries—genomics, proteomics, metabolomics, lipidomics, epigenomics, transcriptomics, and neuronomics—particularly regarding their association with potential endophenotypes and biomarkers for disease (59). Much of this work involves researching endophenotypes as intermediaries of underlying biological causes rather than relying on clinical phenotypes (syndromes), with an example being mismatch negativity (a test of how a brain responds to an unexpected stimulus in a repetitive sequence) as an intermediary marker in schizophrenia through relating glutamate/NMDAR dysfunction and auditory hallucinations (52).

As a result, neuropsychiatry provides a structured framework for linking neural systems to cognitive and behavioral manifestations of illness, offering psychiatrists tools that complement relational and psychotherapeutic approaches in an integrated curriculum.

## Case vignettes

The seven vignettes in Table 3, which are fictional cases created for illustrative purposes only, demonstrate how the above key neuropsychiatric and neuropsychoanalytic concepts could prove useful for understanding different psychiatric presentations. These vignettes are practical examples of how these concepts could also be used pedagogically in psychiatry training. The vignettes illustrate how various clinical scenarios can be approached from an integrated neuropsychiatric and neuropsychoanalytic perspective to take advantage of the full breadth of psychiatric knowledge. Moreover, the formulations are intended to illustrate the value of neuroscience as internal scaffolding, rather than form an explicit basis for psychoeducation.

**TABLE 3:** Case vignettes.

Scenario	Question	Suggested answer
Jack is a 7-year-old boy who has been disruptive and misbehaving at school and at home. He is prone to having explosive outbursts of anger and Jane, his mother, feels threatened by him when she tries to engage in limit and boundary setting. Jane and Jack’s father separated some time ago, and Jane, now a single mother to Jack and a younger sibling, is his only caregiver and feels overwhelmed. Jane believes Jack has a conduct disorder “just like his father,” and says that most of their weekly activities are in response to Jack’s behavior in order to placate him. They have been seeing a child psychotherapist for parent-child interaction therapy, which has focussed on attachment style, parenting, and communication, but after 12 months of this the same problems are persisting. Jack and Jane are referred to a child psychiatrist for an assessment, who diagnoses Jack with ADHD.	How could the psychiatrist integrate clinical neuroscience and psychoanalytic principles to help understand Jack and Jane?	The psychiatrist can hold both developmental neuroscience and relational perspectives. ADHD traits may reflect differences in attentional and arousal regulation systems, while also being shaped by Jack’s early experiences of being criticized and misunderstood. Rather than framing his difficulties as “brain dysfunction,” the psychiatrist can explain to Jack’s mother that his behavior reflects challenges with frustration tolerance and emotional regulation that can improve with support. Psychoeducation can normalize Jack’s difficulties and reduce shame, while also validating Jane’s stress as a parent. Treatment can include behavioral strategies, parental support, possible medication, and a relational stance that helps Jack feel understood and capable, rather than defective.
An outpatient psychiatrist sees a new patient, David, who is 25 years old, and has been having abnormal perceptual experiences, including auditory hallucinations, for more than 6 months. These are distressing to him and are causing him to isolate himself at home. He is convinced of their basis in reality. David lives with his mother and father, who often fight. His mother displays a high degree of expressed emotion and his father is mostly passive and avoids conflict. The psychiatrist diagnoses David with schizophrenia and wants to talk with him and his parents about the diagnosis and to explain the rationale for taking medication to help.	How could the psychiatrist incorporate clinical neuroscience and psychoanalytic principles in this discussion?	The psychiatrist can explain David’s experiences in a way that preserves dignity and reduces fear, emphasizing that psychotic experiences can arise during overwhelming stress and isolation, and that treatment can help restore clarity and safety. The psychiatrist can use knowledge of neural models for schizophrenia to understand heightened threat perception and difficulties with reality-testing, while therapeutically focusing on establishing trust, emotional safety, and gradual collaborative exploration of his beliefs. Medication can be framed as supporting David’s thinking and reducing distress, alongside psychotherapy and strategies to enhance social connection. Curiosity about David’s subjective experience and personal history remains central. To promote adherence with medication, discussions with David and his parents can reference dopaminergic activity in mesolimbic and mesocortical brain circuits to aid understanding of the rationale for prescription.
Felicity is an 18-year-old woman who engages in superficial self-harm by cutting, and whose emotions can rapidly change from feeling well to feeling deep despair and emptiness. She fears abandonment by others and is very emotionally intense. She lives at home with her mother, with whom she has a fractious relationship, especially since her violent father left without warning. She has had no further contact with him. Felicity comes to an Emergency Department and is seen by a psychiatrist, whom she asks for a diagnosis of bipolar disorder, as she says that “all my friends say I have it.” She threatens to hurt herself if she is not “diagnosed properly.”	What clinical neuroscience and neuropsychoanalytic principles may be useful to consider when talking with Felicity?	When speaking with Felicity, the psychiatrist should foreground emotional experience and the therapeutic relationship. Her intense reactions and fears of abandonment can be understood as survival strategies shaped by earlier inconsistent caregiving. The psychiatrist can draw on developmental and affective neuroscience, recognizing that early relational stress can sensitize emotional systems. This can help adopt a non-blaming stance in the psychiatrist, as well as facilitate psychoeducation with Felicity around how, in states of acute stress, she may be predisposed to inhibition of higher-lever cortical functions (i.e., losing access to her “thinking brain”) with regression into lower-level automatic limbic responses (emergence of her “emotional brain”). Although an oversimplification of the neural processes involved, sensitive discussion of such a model may help reduce Felicity’s confusion or shame related to rapid emotional shifts and impulsive actions. Although diagnostic labels should be used cautiously, framing symptoms through a limbic hyperactivity lens may also lessen her insistence on a bipolar label. In the conversation, emphasis should be on validating her feelings, helping her build emotional language and distress-tolerance skills, and exploring her wish to feel understood.
An inpatient child and adolescent psychiatry unit is struggling with staff retention and satisfaction. The unit deals with severely unwell children and young people, many of whom are admitted under the mental health act. They often come from dysfunctional families with high degrees of conflict and trauma. Some staff in the unit have gone on long-term sick leave or threatened to resign in recent months, and there are regular complaints from staff towards the consultant psychiatrist and other managers in the unit. Junior staff often disagree with suggested management plans or complain frequently about various aspects of the patients’ care.	What clinical neuroscience and neuropsychoanalytic principles might help the consultant psychiatrist understand the roles and current functioning of the unit?	Psychoanalytic principles can help understand the complexities of interactions between staff, parents and young people in an inpatient unit, who each bring their own perspectives, projections, and interpretations. Due to the inherent power imbalances within the unit (managers/doctors/mental health act/parents/children), recognizing transference and countertransference principles are an essential component of effective leadership. For example, parents may feel inadequacy and guilt that their child has ended up in a psychiatric unit, but may project this inadequacy and fear as blame and judgment on the nursing staff, who in turn project this onto the unit’s leadership. Many of the ambivalent and disorganized attachment styles within families admitted to the unit become projectively identified by the staff, who then re-enact these behaviors (sick leave, threats to resign) toward management. Recognizing the defenses against underlying emotions and how these play out against existing power structures is crucial to the survival of the unit. Moreover, staff may regress into lower-level automatic limbic responses when feeling persecuted or stressed. The unit may therefore benefit from introducing stress reduction programs or staff reflection groups to mediate limbic activity and encourage top-down processing.
Carly is a 30-year-old woman who was injured in a motor vehicle accident as a passenger in a car driven by her partner. Initial MRI brain showed diffuse axonal injury of the frontal lobes. In the months following the accident, Carly exhibited good recovery, with the exception of frequent episodes of impulsive verbal anger directed at her partner. Six months after the accident, Carly’s partner reported that she appeared depressed, and was less interested in her usual activities. Furthermore, Carly was short-tempered and demonstrated occasional inappropriate rage toward the treating team, whom she believed were “incompetent.”	What neurological, psychiatric and psychoanalytic factors may be considered when formulating Carly’s presentation?	Neurological factors: Traumatic brain injury (TBI) has been associated with depression and apathy. Frontal lobe damage is associated with deterioration of executive functions including attention and working memory. Damage to the orbitofrontal cortex is common with rapid deceleration injuries during car accidents, and can result in failures of inhibitory control including impulsive aggression and emotional lability. Psychiatric factors: The diagnosis of psychiatric illness following TBI requires careful assessment of the aetiology of symptoms common to both, including emotional lability, irritability, apathy, loss of energy, difficulty concentrating, and sleep/appetite disturbances. A differential diagnosis may warrant consideration of the presence of an adjustment disorder, a depressive disorder due to another medical condition, or personality change due to another medical condition (amongst other possibilities). Psychoanalytic factors: Carly’s frustration with her treating clinicians could be a manifestation of an omnipotent defense toward her own cognitive shortcomings. When not enraged, she was noted to lapse into long blank silences, which felt enervating to those around. With time, she could understand that her rage was self-directed, and she felt grief and sadness at the loss of her previous capacities. She also developed awareness that she had repressed a sense of blame towards her partner as the driver at the time of the accident.
A psychiatrist psychotherapist is seeing Bernard, a 55-year-old man, for weekly psychoanalytic psychotherapy following his marriage breakup. During sessions, Bernard will occasionally begin hysterically laughing. The psychotherapist initially believes that the patient’s laughing could be a manifestation of repressed oedipal aggression, or a defense against the sadness and guilt he feels secondary to the marriage breakup. The therapist tries to understand and interpret the laughing in order to help resolve the symptom. However, after a year of therapy, the laughing still persists. The therapist is getting frustrated because they believe Bernard is in deep denial and that the frequent laughing is getting in the way of true therapeutic progress.	What neuropsychiatric or neuropsychoanalytic principles may help the psychotherapist to understand the situation?	Emotional expression changes can reflect alterations in underlying circuits, which coexist with psychological meaning. The laughing could be a manifestation of a more complex and deeply embedded emotion, but after a year of regular therapy, some progress may be expected. Therefore, it may be helpful for the psychiatrist psychotherapist to consider alternative explanations, such as pseudobulbar affect or a tic disorder. Other underlying causes such as an early-onset frontotemporal dementia or a stroke leading to focal or network dysfunction could also be considered. These other causes may be more likely if the patient had a sudden change in personality, such as becoming more irritable, which may have predisposed the marriage breakup. Interpreting changes to his dream phenomenology may also need to be considered in this regard.
Sally, a 35-year-old woman, has had several previous operations due to recurrent abdominal pain, though there has never been any obvious underlying physical cause found. The surgeons continue to offer exploratory surgery as a means of ruling out underlying pathology. The clinical psychologists in the hospital are at odds with the surgeons, suggesting Sally instead suffers from a “functional” pain disorder, which they say is not physical but rather “in her mind.” The psychiatrist in the hospital is asked to provide an opinion on what is contributing to her pain.	Could neuropsychiatric or neuropsychoanalytic insights provide any help to the psychiatrist?	The psychiatrist can validate Sally’s experience by emphasizing that her symptoms are real and distressing, and do not imply that she is “making them up.” Drawing on Solms’ reframing, the clinician can understand her symptoms as arising from disrupted integration between emotional meaning, bodily prediction, and conscious awareness—where overwhelming affect may be expressed through bodily channels when symbolic processing is overburdened. Clinically, the psychiatrist can focus on helping her feel believed, gently exploring stressors and emotional conflicts, and supporting her capacity to link feelings and bodily states over time. Treatment may involve building emotional awareness, grounding techniques, physiotherapy, and psychotherapy focused on affect tolerance and meaning-making, while avoiding invalidating explanations. Moreover, the invasive nature of the surgeon’s repeated actions could be understood in terms of a response to strong levels of projective identification or a reaction to feelings of impotency at being unable to “fix” Sally’s issues. The divide between the psychologists and surgeons could also reflect a splitting defense against this anxiety.

### Discussion of vignettes

These vignettes illustrate a graded approach to integrating neuroscience and psychoanalytic understanding. In some cases (e.g., traumatic brain injury, neurodegenerative disease), neurobiological contributions provide essential clinical guidance. In others, neuroscience informs the clinician’s conceptual stance, rather than altering therapeutic technique or psychoeducation. Although a formulation must represent a shared understanding between psychiatrist and patient, particular care must be given to whether a neuroscientific explanation is included and, if so, ensuring that it presented in a format commensurate with the patient’s level of health literacy. If in doubt, maintaining the focus of therapeutic discussions on supporting empathy, reducing blame, and guiding treatment—rather than excessively esoteric neuroscience-based explanations—allows formulation to remain valuable to the patient and retain emphasis on subjective meaning. This reflects the non-reductive dual-aspect position emphasized throughout the paper: neuroscience contributes where it genuinely aids understanding or planning, while the relational, symbolic, and developmental dimensions remain central to clinical care.

## Conclusion

A psychiatric clinical neuroscience curriculum that incorporates perspectives from neuropsychoanalysis and neuropsychiatry would be a valid and valuable addition to existing psychiatric training. Key perspectives described included a focus on brain networks, levels of consciousness, neurodevelopment, affective neuroscience, and mind–brain philosophy. Importantly, concepts from neuropsychoanalysis may particularly help to strengthen the utility of psychotherapy and psychoanalysis within psychiatry. Broadly, the concepts highlighted in this paper could inform psychiatric curricula through their use in lecture material, online teaching modules, bedside teaching, psychotherapy training for psychiatrists, and case formulation discussions.

## Data Availability

The original contributions presented in this study are included in this article/supplementary material, further inquiries can be directed to the corresponding author.

## Footnotes

1
https://nncionline.org/at-a-glance/

2
https://www.sfn.org/initiatives/neuronline

3
https://www.genes2cognition.org/

4
https://www.fens.org/publications/resources
